# Pine ( *Pinus densiflora* ) needle extract could promote the expression of PCNA and Ki-67 after partial hepatectomy in rat ^1^

**DOI:** 10.1590/s0102-865020190060000006

**Published:** 2019-08-19

**Authors:** Gyeong Seok Lee, Hyeon Gung Yang, Ji Hun Kim, Young Mo Ahn, Man Deuk Han, Wan Jong Kim

**Affiliations:** I Department of Life Science and Biotechnology , Soonchunhyang University , Asan , Korea . Conception and design; acquisition, analysis and interpretation of data; technical procedures; histopathological examinations; statistics analysis; manuscript writing, final approval.; II Department of Life Science and Biotechnology , Soonchunhyang University , Asan , Korea . Conception and design; acquisition, analysis and interpretation of data; technical procedures; histopathological examinations; statistics analysis; manuscript preparation and writing, final approval.; III Department of Life Science and Biotechnology , Soonchunhyang University , Asan , Korea . Acquisition of data, manuscript preparation, final approval.; IV PhD, Department of Life Science and Biotechnology , Soonchunhyang University , Asan , Korea . Acquisition of data, histopathological examinations, critical revision, final approval.; V PhD, Professor, Department of Life Science and Biotechnology , Soonchunhyang University , Asan , Korea . Analysis and interpretation of data, histopathological examinations, critical revision, final approval.; VI PhD, Professor, Department of Life Science and Biotechnology , Soonchunhyang University , Asan , Korea . Conception and design of the study, histopathological examinations, manuscript writing, critical revision, final approval.

**Keywords:** Liver Regeneration, Hepatectomy, Proliferating Cell Nuclear Antigen, Hepatocytes, Rats

## Abstract

**Purpose:**

To investigate the effects of pine needle extract (PNE) on the expression of proliferating cell nuclear antigen (PCNA) and Ki-67 during liver regeneration induced by 70% partial hepatectomy (PH) in rat.

**Methods:**

Forty-eight male rats (SD, 7 weeks) had surgery (70% PH). They were randomly divided into two groups. PH + PNE group was only provided PNE diluted in water (10%) for drinking and PH group was provided water from 5 days before surgery to the time of sacrifice. PNE was made by pressing and filtering. Animals were sacrificed at 12h, 24h, 36h, 60h, 84h, 168h after PH, respectively. The expressions of PCNA and Ki-67 were determined as proliferation indices.

**Results:**

Immunohistochemistry turned out to increase the expression of PCNA and Ki-67. PCNA expression of PH+PNE group increased up to twice of that of PH group. Western blot also seemed to increase the PCNA expression. These results indicated the promotion of cell proliferation in liver tissue and hepatic regeneration.

**Conclusions:**

Pine needle extract stimulates the expression of some mitotic proteins during liver regeneration induced by 70% PH in rats. It suggests that administration of pine needle extract could accelerate the liver regeneration after partial hepatectomy.

## Introduction

The liver plays a crucial role in metabolism that involves blood glucose regulation, protein synthesis, bile production, detoxification, and urea production. It also maintains metabolic homeostasis and harmony in the body, and acts in cooperation with many organs. In rats, median and left lateral lobes of liver comprise about 70% of total liver volume. The main type of hepatic cell is hepatocyte. The liver has the unique ability to regenerate after injury, and the term ‘liver regeneration’ is generally used in scholarly articles. It has been more precisely termed ‘compensatory hyperplasia’ of liver tissue ^1 - 3^ . This phenomenon involves rapid growth of the liver tissue after injury induced tissue loss or damage.

Liver regeneration can be induced by 70% partial hepatectomy (PH), a surgery that removes two-thirds of the liver. This surgical procedure was first established by Higgins and Anderson ^4^ . After PH, the residual parenchyma of liver quickly grows to restore the original mass and to meet metabolic needs ^2 , 5 , 6^ . This surgical model has certain advantages: the livers of all experimental animals are damaged equally, and the remnant liver tissues are not damaged. In addition, the model allows researchers to accurately identify the time at which regeneration begins. After PH, hepatic cells switch from a quiescent to a cycling state, which is generally ascribed to the action of cytokine-dependent signaling pathways. Hepatocyte growth factor (HGF), transforming growth factor alpha (TGF-α), and epidermal growth factor (EGF) have been recognized as hepatic mitogens that allow hepatic cells to overcome the G _1_ restriction point ^7^ . Other organs also participate in regeneration, and cooperative signals come from the pancreas, duodenum, salivary gland, thyroid gland, and adrenal gland ^3 , 8 , 9^ . Generally, DNA replication for proliferation in hepatocytes starts at 12h and peaks at 24h. Other type of hepatic cells (Kupffer cell, Ito cell and endothelial cell) proliferate later ^1 , 3^ . In total, about 1.6 cycles of replication are necessary to fully restore the liver ^3^ . However, the regeneration does not restore the original anatomical structure of the hepatic lobes ^10^ . Recent studies have shown that silymarin can accelerate the liver regeneration ^11^ . It was also reported that innate immune responses are related to regeneration process ^12^ .

The pine tree ( *Pinus densiflora* ) is a needle-leaf tree distributed in Eastern Asia, including Korea, China, Japan, and Russia. The needles have conventionally been used in traditional Oriental medicine for gastroenteric troubles, hemorrhage, and hypertension ^13^ . Recently, it was reported that pine needles have anti-oxidant, anti-mutagenic, anti-tumor, anti-bacterial, anti-inflammatory and memory enhancing activities ^14 - 18^ . Moreover, it also has been reported that bark and pollen of pine trees have anti-inflammatory and analgesic activities ^19 , 20^ . Several chemicals in pine needles were identified: α-pinene, β-pinene, camphene, β-phellandrene, citronellol, and β-caryophyllene ^21^ . The components of pine needles can differ depending on region, climate, and other geographical and environmental features.

In the present study, we investigated the proliferating effects of the pine needle extract (PNE) on liver regeneration induced by 70% PH in rats. It was fed *ad libitum* to rats. We focused on the proliferation of hepatic cells and expressions of some protein involving proliferating cell nuclear antigen (PCNA) and Ki-67.

## Methods

### Animals and treatments

All protocols for animal experimentation were approved by the Institutional Animal Care and Use Committee of Soonchunhyang University (permission number: SCH16-0021) and conducted in conformity with the Guide for the Care and Use of Laboratory Animals (NIH Publications, No. 8023).

Male Sprague–Dawley rats (body weight; 200 ± 10g, 7 weeks old, SPF) were used. They were acclimatized before the beginning of the experiment and housed in an environmentally controlled room at 22°C, with a 12h light/dark cycle, 60% relative humidity, and unrestricted access to standard food and water.

To establish the 70% partial hepatectomized rat model, we carried out 70% partial hepatectomy (PH) according to the procedure of Higgins under isoflurane (Piramal Critical Care, Bethlehem, PA, USA) inhalation anesthesia ^4^ . After PH, all animals were relaxed in new comfortable bedding under a warm lamp. Hepatectomized rats were randomly divided into two groups: PH + PNE group (experimental group, 24 rats) was given pine needle extract (PNE) diluted in water (10%) instead of water, and the other group, PH group (control group, 24 rats) was given water for drinking. All rats had unrestricted access to drinking fluids. PNE was provided to rats in the PH + PNE group from 5 days before PH to the time of sacrifice. In the pretest, we determined the rat’s approximate daily liquid consumption. Each rat drank averagely 25 mL of PNE diluted in water (10%) per day; therefore, each rat consumed 2.5 mL of PNE per day. Animals were sacrificed at 12h, 24h, 36h, 60h, 84h, 168h after PH, respectively. After sacrifice, regenerated liver including the right lateral lobe and the caudate lobe and blood were collected for analysis. After the recording of the regenerated liver weight, tissue from the right lateral lobe of each animal was used for further analysis.

### Preparation of pine needle extract

Pine needle extract (PNE) used in experiment was provided by Dongyang E&P Company (Seosan, Chungcheongnam-do, Korea). Pine ( *Pinus densiflora* ) needles were collected from Seosan, Chungcheong nam-do, Korea in November 2012. Collected pine needles were washed and chopped and then pressed to obtain extract at 4°C. Obtained liquid extract was filtered using a Whatman filter paper and stored in a refrigerator at 4°C. The extract was diluted to 10% concentration with distilled water before use.

### Immunohistochemical analysis for PCNA and Ki-67

For light microscopy, liver tissues were cut and fixed in 10% neutral buffered formalin. Tissues were embedded in paraffin and cut using a rotary microtome (RM2235; Leica Biosystems, Wetzlar, Hessen, Germany). For immunohistochemistry, antigen retrieval step was performed by the heat-induced epitope retrieval method with sodium citrate buffer (10 mM sodium citrate, 0.05% Tween 20, pH 6.0). Sections were treated with 3% hydrogen peroxide and 10% bovine serum albumin (BSA), then incubated with primary antibodies against PCNA (1:1,000, 4°C, overnight; Abcam, Cambridge, Cambridgeshire, UK), Ki-67 (1:200, room temperature, 90 min; Abcam, Cambridge, Cambridgeshire, UK). To verify the specificity of the immunohistochemical reactions, we prepared negative controls without primary antibody treatment. A secondary antibody conjugated with horseradish peroxidase and DAB (Dako REAL™ EnVision™ Detection System; Dako, Glostrup, Region Midtjylland, Denmark) were applied. All immunohistochemistry steps were carried out in a humidified chamber. To count unstained cells, we performed counter staining with hematoxylin. To assess the expression of PCNA and Ki-67, we photographed liver tissues containing the central vein with light microscope (BX43; Olympus, Tokyo, Tokyo, Japan) and digital camera (eXcope T300; DIXI Science, Daejeon, Korea). Microphotographs were randomly selected for counting. The number of hepatocytes with positive reactions against PCNA and Ki-67 were manually counted.

### Western blot analysis for PCNA

Liver tissues were homogenized with a homogenizer (DIAX 100; Heidolph, Schwabach, Freistaat Bayern, Germany) with protein extraction solution (PRO-PREP ^™^ ; iNtRON BIOTECHNOLOGY, Seongnam, Gyeonggi-do, Korea) on ice. Proteins were determined with the bicinchoninate (BCA) assay using kit (Pierce ^™^ BCA Protein Assay Kit, #23227; Thermo Fisher Scientific, Rockford, IL, USA). A total of 30 μg protein per lane was run on a 12% sodium dodecyl sulfate polyacrylamide gel at 200 V, 100 mA and transferred to a polyvinylidene difluoride (PVDF) membrane (Immobilon ^®^ ; Millipore, Burlington, MA, USA) at 100 V, 200 mA. Membranes were blocked with 3% skim milk or 3% BSA and incubated with primary antibodies against PCNA (1:3,000, monoclonal antibody; Abcam, Cambridge, Cambridgeshire, UK), and β-actin (1:6,000, monoclonal antibody; Sigma-Aldrich, St. Louis, MO, USA) at 4°C, overnight. After washing, membranes were incubated with an HRP-conjugated secondary antibody (1:5,000; Thermo Fisher Scientific, Rockford, IL, USA). After washing, the targeted protein bands were detected using enhanced chemiluminescence (Western Bright ECL Spray; Advansta, Menlo Park, CA, USA). Signals were recorded using G:BOX iChemi XL (Syngene, Cambridge, Cambridgeshire, UK).

### Statistical analysis

Data were expressed as mean ± standard deviation (SD) from four independent experiments. Data were analyzed by *t* -test using PASW Statistics 18 statistics program (IBM, Chicago, IL, USA). A *p* value of less than 0.05 was considered statistically significant.

## Results

### Immunohistochemistry for PCNA and Ki-67

**Figure 1:**
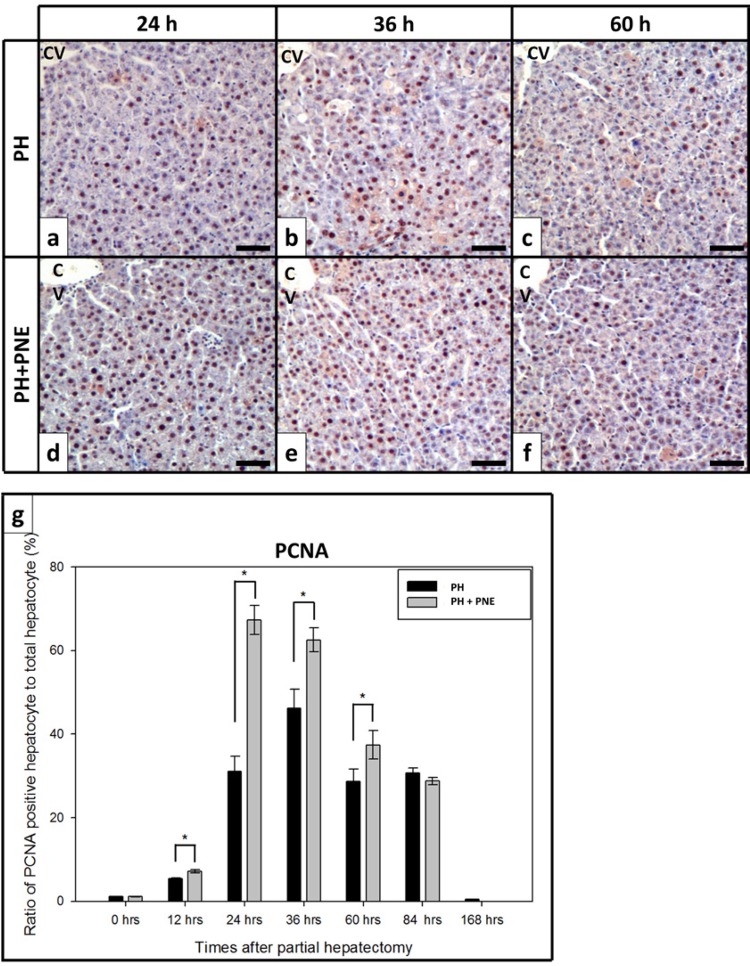
– Microphotographs of immunohistochemistry for PCNA. Scale bar indicates 100 μm. (n=4, *: p<0.05, CV: central vein) PCNA expression of PH + PNE groups was more increased than that in the PH group.

**Figure 2:**
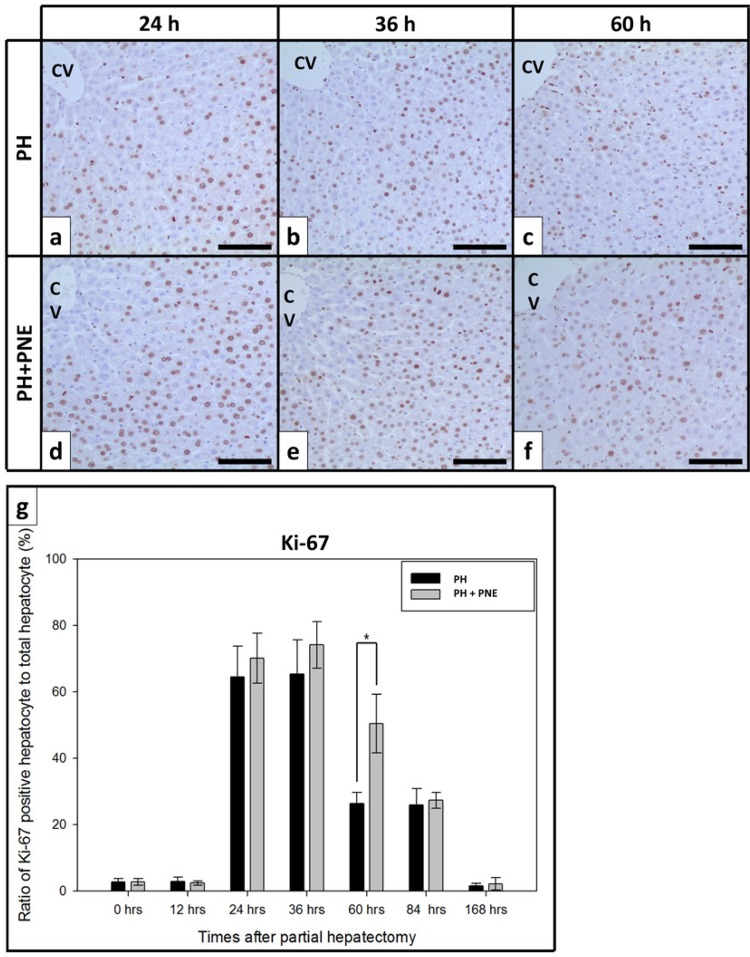
Microphotographs of immunohistochemistry for Ki-67. Scale bar indicates 100 μm. (n=4, *: p<0.05, CV: central vein) After 24h, positively reacted hepatocytes for Ki67 were more increased in PH + PNE groups.

To investigate the proliferation of hepatocytes, we carried out immunohistochemistry for proliferating cell nuclear antigen (PCNA) and Ki-67 ( Figs. 1 and 2 ). PCNA, which is a cofactor of DNA polymerase, was prevalent in the nuclei of proliferating hepatocytes. We counted the number of hepatocytes in photomicrographs. The ratio of positively reacted hepatocytes/total hepatocytes increased after surgery ( Fig. 1 ). The ratio dramatically increased at 24h after PH. In the PH group, there was a 30% increase, whereas in the PH + PNE group, there was a 70% increase. During the regeneration, the ratio of the PH + PNE group was greater than that in the PH group. At 168h after PH, the ratio decreased to a normal value in both groups. We also carried out analysis for Ki-67, a specific marker of proliferation, and it was also present in the nuclei of proliferating cells. Ki-67 expression also increased like PCNA expression during regeneration. The proliferating hepatocyte (positive reacted for Ki-67)/total hepatocyte ratio was greater than that in PH group.

### Western blot analysis for PCNA

**Figure 3:**
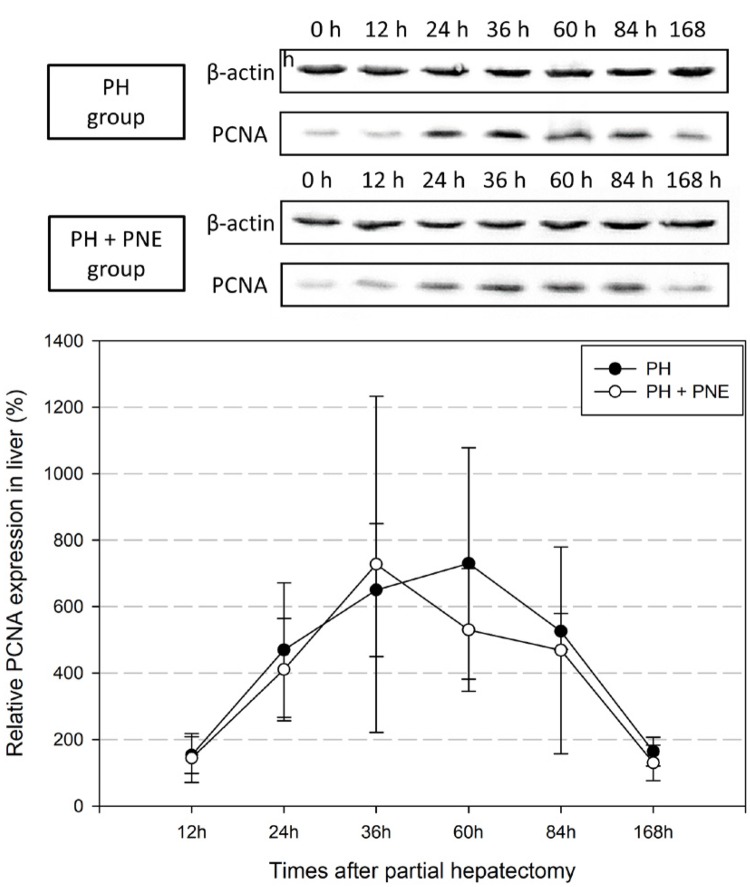
Western blot for PCNA. PCNA expression shows total hepatic proliferation. It was slightly altered with PNE. Beta-actin was used as a loading control protein.

The pattern of PCNA expression was altered during liver regeneration ( Fig. 3 ). There could be seen the proliferation of total hepatic cells including hepatocytes, Kupffer cells and endothelial cells. The β-actin was used as a loading control protein. The density ratio of the PH + PNE group peaked at 36h after surgery and then decreased, while the PH group showed the peak at 60h. After 168h, PCNA expression in both groups had returned to a normal value.

## Discussion

In the present study, we investigated the effects of pine needle extract (PNE) on liver regeneration induced by 70% partial hepatectomy (PH) in a rat model. We focused on proliferation of hepatocytes and expression of proliferating cell nuclear antigen (PCNA) and Ki-67 protein. During regeneration, quiescent hepatocytes undergo replication and then return to a non-proliferative state. This process is regulated by certain growth factors, including HGF (hepatocyte growth factor), EGF (epidermal growth factor), TNF-α (tumor necrosis factor alpha), VEGF (vascular endothelial growth factor), TGF-β (transforming growth factor beta) and other cytokines ^22 - 25^ .

Microscopically, we observed the collapse and restoration of the hepatic structure and cellular division during regeneration. Immunohistochemistry showed altered PCNA and Ki-67 expression with PNE treatment during liver regeneration. PCNA and Ki-67 expression of PH + PNE group are more increased than that in the PH group. We counted only hepatocyte for immunohistochemistry. Thus, immunohistochemical results revealed increase of proliferation of hepatocytes (hepatic parenchyma). It also means that PNE has mitotic activity. Western blot analysis of remnant liver tissue also showed altered expression of PCNA. The expression of the PH + PNE group peaked earlier than that of the PH group. It can show a different point related to the proliferation of all types of hepatic cells including hepatocytes, Ito cells, Kupffer cells and endothelial cells. Thus, the PNE administration could stimulate hepatic proliferation during liver regeneration process.

Many natural products, drugs and chemicals were known as the materials to promote the liver regeneration after partial hepatectomy, as silymarin, nebivolol, schisandrol B ^11 , 12 , 26 , 28^ . They showed anti-oxidative activity, cytoprotective activity, mitotic effects and other metabolic changes during liver regeneration. In addition, some growth factors can promote liver regeneration ^22^ . Anti-oxidative activities are a well known feature of phenolic compounds. The pine needle has many phenolic compounds such as pinene ^28^ . Thus, PNE can affect liver regeneration through reducing the oxidative stress created by the liver regeneration process. We think that these anti-oxidative feature and other effectiveness of PNE may promote liver regeneration.

In the present study, we investigated the proliferative effects of PNE on liver regeneration induced by 70% partial hepatectomy in rat. We found that PNE administration could increase the expression of PCNA and Ki-67. These results suggest that PNE stimulated the liver regeneration after partial hepatectomy in rats.

## Conclusion

Fermented pine ( *Pinus densiflora* ) needle extract stimulated some mitotic proteins as proliferating cell nuclear antigen and proliferation of hepatic cells during liver regeneration induced by 70% partial hepatectomy in rats.

## Footnotes

1 Research performed at the Cell Biology Laboratory, Department of Life Science and Biotechnology, Soonchunhyang University, Asan, Republic of Korea.

## References

[1] Fausto N, Riehle KJ (2005). Mechanisms of liver regeneration and their clinical implications. J Hepatobiliary Pancreat Surg.

[2] Rychtrmoc D, Libra A, Buncek M, Garnol T, Cervinková Z (2007). Studying liver regeneration by means of molecular biology: how far we are in interpreting the findings?. Acta Me.

[3] Taub R (2004). Liver regeneration: from myth to mechanism. Nat Rev Mol Cell Biol.

[4] Higgins GM, Anderson RM (1931). Restoration of the liver of the white rat following partial surgical removal. Arch Pathol.

[5] Aller MA, Arias N, Prieto I, Agudo S, Gilsanz C, Lorente L, Arias JL, Arias J (2012). A half century (1961–2011) of applying microsurgery to experimental liver research. World J Hepatol.

[6] Rodríguez G, Lorente L, Durán HJ, Aller MA, Arias J (1999). A 70% hepatectomy in the rat using a microsurgical technique. Int Surg.

[7] Loyer P, Cariou S, Glaise D, Bilodeau M, Baffet G, Guguen-Guillouzo C (1996). Growth factor dependence of progression through G1 and S phases of adult rat hepatocytes in vitro: evidence of mitogen restriction point in mid-late G1. J Biol Chem.

[8] Červinková Z, Šimek J, Trojovská V (1984). Effect of triiodothyronine or etiroxate on DNA synthesis in intact and regenerating liver. Physiol Bohemoslov.

[9] Tarlá MR, Ramalho FS, Ramalho LN, Silva TC, Brandäo DF, Ferreira J, Silva OC, Zucoloto S (2006). A molecular view of liver regeneration. Acta Cir Bras.

[10] Mao SA, Glorioso JM, Nyberg SL (2014). Liver regeneration. Transl Res.

[11] Han SK, Lee GS, Yoo TK, Yang HG, Kim JH, Ahn YM, Han MD, Kim WJ (2016). Administration of silymarin could promote the expression of proliferating cell nuclear antigen during liver regeneration induced by partial hepatectomy in rats. Acad J Biotechnol.

[12] Chen GW, Zhang MZ, Zhao LF, Xu CS (2006). Expression patterns and action analysis of genes associated with physiological responses during rat liver regeneration: innate immune response. World J Gastroenterol.

[13] Kwon JH, Kim JH, Choi SE, Park KH, Lee MW (2010). Inhibitory effects of phenolic compounds from needles of Pinus densiflora on nitric oxide and PGE2 production. Arch Pharm Res.

[14] Hwang YJ, Wi HR, Kim HR, Park KW, Hwang KA (2014). Pinus densiflora Sieb. et Zucc. alleviates lipogenesis and oxidative stress during oleic acid-induced steatosis in HepG2 cells. Nutrients.

[15] Kwak CS, Moon SC, Lee MS (2006). Antioxidant, antimutagenic, and antitumor effects of pine needles ( Pinus densiflora ). Nutr Cancer.

[16] Won SB, Jung GY, Kim J, Chung YS, Hong EK, Kwon YH (2013). Protective effect of Pinus koraiensis needle water extract against oxidative stress in HepG2 cells and obese mice. J Med Food.

[17] Lee JS, Kim HG, Lee HW, Kim WY, Ahn YC, Son CG (2017). Pine needle extract prevents hippocampal memory impairment in acute restraint stress mouse model. J Ethnopharmacol.

[18] Lee JS, Kim HG, Lee HW, Han JM, Lee SK, Kim DW, Saravanakumer A, Son CG (2015). Hippocampal memory enhancing activity of pine needle extract against scopolamine-induced amnesia in a mouse model. Sci Rep.

[19] Choi EM (2007). Antinociceptive and antiinflammatory activities of pine ( Pinus densiflora ) pollen extract. Phytother Res.

[20] Ince I, Yesil-Celiktas O, Karabay-Yavasoglu NU, Elgin G (2009). Effects of Pinus brutia bark extract and Pycnogenol® in a rat model of carrageenan induced inflammation. Phytomedicine.

[21] Koukos PK, Papadopoulou KI, Patiaka DT, Papagiannopoulos AD (2000). Chemical composition of essential oils from needles and twigs of balkan pine ( Pinus peuce Grisebach) grown in Northern Greece. J Agric Food Chem.

[22] Böhm F, Köhler UA, Speicher T, Werner S (2010). Regulation of liver regeneration by growth factors and cytokines. EMBO Mol Med.

[23] Michalopoulos GK (2007). Liver regeneration. J Cell Physiol.

[24] Stolz DB, Mars WM, Petersen BE, Kim TH, Michalopoulos GK (1999). Growth factor signal transduction immediately after two-thirds partial hepatectomy in the rat. Cancer Res.

[25] Tarlá MR, Ramalho FS, Ramalho LN, Silva TC, Brandäo DF, Ferreira J, Silva O C, Zucoloto S (2006). Cellular aspects of liver regeneration. Acta Cir Bras.

[26] Sumer F, Colakglu MK, Ozdemir Y, Ozsay O, ílter O, Bostanci EB, Akoglu M (2015). Effect of nebivolol on liver regeneration in an experimental 70% partial hepatectomy model. Asian J Surg.

[27] Li X, Sun J, Fan X, Guan L, Li D, Zhou Y, Zeng X, Chen Y, Zhang H, Xu L, Jiang F, Huang M, Bi H (2018). Schisandrol B promotes liver regeneration after partial hepatectomy in mice. Eur J Pharmacol.

[28] Kim JS (2018). Evaluation of In vitro antioxidant activity of the water extract obtained from dried pine needle ( Pinus densiflora ). Prev Nutr Food Sci.

